# Comparison of Diagnostic and Triage Accuracy of Ada Health and WebMD Symptom Checkers, ChatGPT, and Physicians for Patients in an Emergency Department: Clinical Data Analysis Study

**DOI:** 10.2196/49995

**Published:** 2023-10-03

**Authors:** Hamish Fraser, Daven Crossland, Ian Bacher, Megan Ranney, Tracy Madsen, Ross Hilliard

**Affiliations:** 1 Brown Center for Biomedical Informatics The Warren Alpert Medical School of Brown University Providence, RI United States; 2 Department of Health Services, Policy and Practice Brown University School of Public Health Providence, RI United States; 3 Department of Epidemiology Brown University School of Public Health Providence, RI United States; 4 School of Public Health Yale University New Haven, CT United States; 5 Department of Emergency Medicine The Warren Alpert Medical School of Brown University Providence, RI United States; 6 Department of Internal Medicine Maine Medical Center Portland, ME United States

**Keywords:** diagnosis, triage, symptom checker, emergency patient, ChatGPT, LLM, diagnose, self-diagnose, self-diagnosis, app, application, language model, accuracy, ChatGPT-3.5, ChatGPT-4.0, emergency, machine learning

## Abstract

**Background:**

Diagnosis is a core component of effective health care, but misdiagnosis is common and can put patients at risk. Diagnostic decision support systems can play a role in improving diagnosis by physicians and other health care workers. Symptom checkers (SCs) have been designed to improve diagnosis and triage (ie, which level of care to seek) by patients.

**Objective:**

The aim of this study was to evaluate the performance of the new large language model ChatGPT (versions 3.5 and 4.0), the widely used WebMD SC, and an SC developed by Ada Health in the diagnosis and triage of patients with urgent or emergent clinical problems compared with the final emergency department (ED) diagnoses and physician reviews.

**Methods:**

We used previously collected, deidentified, self-report data from 40 patients presenting to an ED for care who used the Ada SC to record their symptoms prior to seeing the ED physician. Deidentified data were entered into ChatGPT versions 3.5 and 4.0 and WebMD by a research assistant blinded to diagnoses and triage. Diagnoses from all 4 systems were compared with the previously abstracted final diagnoses in the ED as well as with diagnoses and triage recommendations from three independent board-certified ED physicians who had blindly reviewed the self-report clinical data from Ada. Diagnostic accuracy was calculated as the proportion of the diagnoses from ChatGPT, Ada SC, WebMD SC, and the independent physicians that matched at least one ED diagnosis (stratified as top 1 or top 3). Triage accuracy was calculated as the number of recommendations from ChatGPT, WebMD, or Ada that agreed with at least 2 of the independent physicians or were rated “unsafe” or “too cautious.”

**Results:**

Overall, 30 and 37 cases had sufficient data for diagnostic and triage analysis, respectively. The rate of top-1 diagnosis matches for Ada, ChatGPT 3.5, ChatGPT 4.0, and WebMD was 9 (30%), 12 (40%), 10 (33%), and 12 (40%), respectively, with a mean rate of 47% for the physicians. The rate of top-3 diagnostic matches for Ada, ChatGPT 3.5, ChatGPT 4.0, and WebMD was 19 (63%), 19 (63%), 15 (50%), and 17 (57%), respectively, with a mean rate of 69% for physicians. The distribution of triage results for Ada was 62% (n=23) agree, 14% unsafe (n=5), and 24% (n=9) too cautious; that for ChatGPT 3.5 was 59% (n=22) agree, 41% (n=15) unsafe, and 0% (n=0) too cautious; that for ChatGPT 4.0 was 76% (n=28) agree, 22% (n=8) unsafe, and 3% (n=1) too cautious; and that for WebMD was 70% (n=26) agree, 19% (n=7) unsafe, and 11% (n=4) too cautious. The unsafe triage rate for ChatGPT 3.5 (41%) was significantly higher (*P*=.009) than that of Ada (14%).

**Conclusions:**

ChatGPT 3.5 had high diagnostic accuracy but a high unsafe triage rate. ChatGPT 4.0 had the poorest diagnostic accuracy, but a lower unsafe triage rate and the highest triage agreement with the physicians. The Ada and WebMD SCs performed better overall than ChatGPT. Unsupervised patient use of ChatGPT for diagnosis and triage is not recommended without improvements to triage accuracy and extensive clinical evaluation.

## Introduction

Accurate diagnosis is a key part of effective patient care, but misdiagnosis is common and can be harmful to patients [1]. Misdiagnosis can lead to delayed recognition of the true condition, which can misdirect the clinical history-taking, examination, and investigation. Diagnostic decision support systems have been developed for over 50 years, initially to support physicians [2-4]. However, over the last decade, their use has extended to direct and unsupervised use by patients with the development of diagnostic apps termed symptom checkers (SCs). Evidence from evaluation studies performed to date has shown that SCs have highly variable performance when tested with case vignettes (which typically include demographic data, some past medical history, and current symptoms). The best-performing systems have shown diagnostic and triage performance close to that of physicians using the same data, although poorer-performing systems have been shown to have lower accuracy than physicians or even patients tested on the same case vignettes [5-7].

Large language models (LLMs) are a new type of artificial intelligence technology designed primarily to predict the next words and phrases given some initial text. LLMs utilize neural networks that are inspired by neural structures seen in the human brain [8]. These networks are initially provided with certain tokens, which can consist of data points such as words or phrases, that allow the LLMs to “chunk” and analyze vast amounts of training data. As the LLM analyzes the data, patterns and associations between various tokens and other data contained in the training set build a map in which relationships between data points are quantified by differently weighted parameters. Once the model is appropriately trained and reliably produces the desired output, the outputs are reviewed and rated for accuracy by experts in the context the LLM is to be used, a process referred to as reinforcement learning with human feedback (RLHF) [9]. These ratings are then fed back to the LLM to improve the model’s accuracy for a specific objective. LLMs have also demonstrated in-context learning, in which an existing model can complete a task after being prompted with a small number of examples, despite not originally being trained on these data [10].

GPT, or Generative Pre-trained Transformer (OpenAI Inc, San Francisco, CA), is a type of LLM that uses a transformer neural network with hundreds of billions of parameters to output human-like dialogue [11]. A recent version, GPT-3, was trained with a very large corpus of 570 Gigabytes of text collected from the internet. A newer version, GPT-3.5, has been shown to be capable of a wide range of tasks in different fields, including law and medicine. This model was linked to a chatbot to create ChatGPT 3.5, a version with which internet users can directly interact. Initial studies have shown that ChatGPT is capable of answering a wide range of questions about medicine and health. These include providing advice to patients on heart disease [12], performing tasks to support primary care physicians [13], and passing medical board exams based on performance in answering sample questions [14]. Studies have also shown that ChatGPT is capable of producing differential diagnoses when presented with medical data such as case summaries [15-17]. As with other uses of ChatGPT, there have been many examples reported of high accuracy or performance on medical tasks along with many examples of it outputting made-up data or “hallucinations” [18]. In many professional situations, an expert user can review the output of a system like ChatGPT and correct such errors; however, this is not generally the case if patients use the system directly. This situation is analogous to the use of SC apps, where poorly designed and evaluated SCs could put patients at risk [19]. ChatGPT is currently available (July 2023) in 2 versions: the original, publicly available 3.5 version and a subscription-based 4.0 version trained on an even larger set of data. It has not been reported whether either model has undergone specific RLHF for medicine or health care.

**Figure 1:**
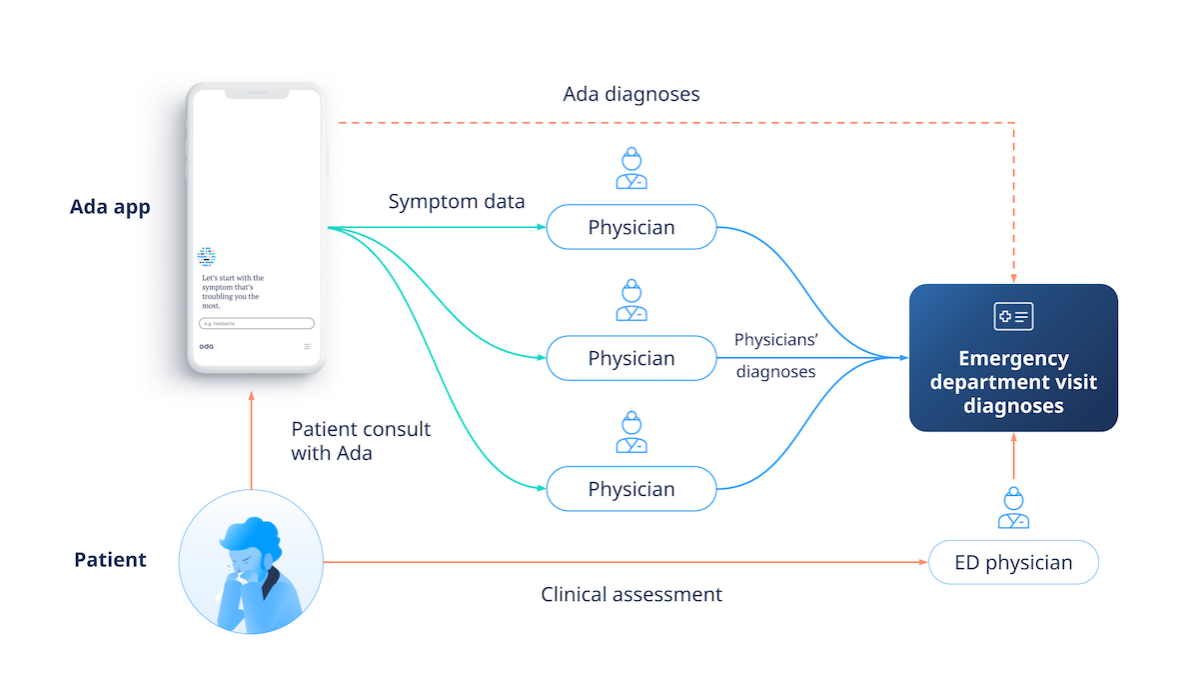
Data collection and flow in the Rhode Island Hospital emergency department (ED) study [20].

In a previous study [20], we evaluated a widely used SC from Ada Health, which in 2022 had been used by 11 million users carrying out 23 million health assessments [21]. The Ada app includes a chatbox functionality for eliciting a clinical history and a diagnostic and triage algorithm that has been extensively tested and updated based on preclinical and clinical testing. The system is based on a Bayesian network, which was built using extensive data from published clinical studies and input from expert clinicians. As with most available SCs, Ada is not based on a machine learning approach [7]. Ada has supported many clinical evaluation studies in-house and assisted independent researchers, providing extensive published data on its usability and its diagnostic and triage performance [6,7,20,22-24]. In the previous study, the Ada SC was used by patients waiting for clinical assessment in the emergency department (ED) of Rhode Island Hospital (RIH), Rhode Island, USA. The diagnoses of Ada were compared to the physicians’ visit notes after they had seen the patient. The symptom data collected by Ada were then blindly reviewed by 3 independent ED physicians who provided their own diagnosis and triage results and then critiqued the Ada results. Triage is the process of assessing the risk of clinical deterioration of a patient and using this to prioritize care, which is typically carried out by a nurse or physician using a standard system soon after a patient’s arrival at an ED. Figure 1 shows the study design and data collection and flow. Overall, Ada performed well with diagnostic accuracy close to the physician reviewers. The unsafe triage rate of 15% (with at least 2 of 3 physician reviewers agreeing) was similar to or better than that found in several studies of nurse triage [20].

While ChatGPT has been shown to have surprisingly good diagnostic accuracy on a range of clinical case descriptions, examples reported to date appear to use standardized case vignettes rather than real patient consultations. For example, Levine et al [15] tested ChatGPT on the 48 standard case vignettes that were previously used for a study of patients’ performance in diagnosis and triage after reading these vignettes. The results showed high diagnostic accuracy, whereas triage accuracy was moderately good but no better than that of the patients overall (see the Discussion for more details).

Our objective in this study was to evaluate the diagnostic and triage accuracy of ChatGPT 3.5 and 4.0 using the real patient data collected by the Ada SC in the ED and the actual diagnoses from the ED physicians who saw the patients. We also evaluated the most commonly used SC app in the United States, WebMD [25], in an identical fashion for comparison.

## Methods

### Data Source

The data for this study came from a previous study performed at the RIH ED [20]. Patients in an assessment room waiting to see an ED physician were (1) recruited by a research assistant (RA), (2) completed a consent form, (3) used the Ada SC app on an iPad to self-report symptoms, and then (4) completed a short user survey in REDCap [26]. Patients with acute or serious medical conditions presenting to an ED were deemed eligible for inclusion in the study. In addition, the patients had to be English-speaking; aged ≥18 years; presenting for emergency evaluation of a medical (ie, nontrauma and nonmental health) problem; and deemed by the triage nurse to not be critically ill, defined as an Emergency Severity Index (ESI) score [27] of 2-5. Those with an ESI score of 1 were excluded. The diagnosis and triage results generated by Ada were sent by secure email to the study team and were not shown to the patient or the treating physician. Patients had to complete the study prior to being seen by the ED physician and were then given a US $20 gift card. Information on the final diagnoses from the ED physician’s assessment was extracted by an RA from the EPIC electronic health record (EHR).

### Study Design

In analysis of the prior study [20], after the completion of patient recruitment, the clinical data from Ada (age; sex; history of heart disease, diabetes, and hypertension; current pregnancy; and presenting symptoms and follow-up questions, in the form of a list of symptoms reported as present or absent) were reviewed by 3 independent ED physicians who had not seen the patient. The physicians were asked to provide 3 possible diagnoses and an appropriate triage level (ranging from ED visit to home care). They were also asked to critique the Ada diagnoses and triage suggestions. Overall, 30 cases had sufficient data for diagnostic analysis and 37 cases had sufficient data for triage analysis. A list of all 40 case presentations and diagnoses and an example of the symptom data collected by Ada are available as appendices from Fraser et al [20].

The diagnoses of Ada and of the independent physicians were compared to the final diagnoses from the ED physician. The process of data collection along with the diagnosis and triage comparison method were based on the previous study from Fraser et al [20], as shown in Figure 1. In summary, (1) the Ada app collects the *demographic and symptom data*, (2) these data are later reviewed by 3 independent (ED) physicians, and (3) the *Ada diagnoses* and the 3 *independent physicians’ diagnoses* are compared to the *ED visit diagnoses* recorded in the EHR by the ED physician who carried out the *clinical assessment*. The primary metric for diagnostic accuracy was the percentage of cases where at least one of the Ada or independent physicians (the diagnostic agent) diagnoses matched at least one of the treating physicians’ diagnoses (this could also be defined as the sensitivity of the diagnostic agent for at least one of the final diagnoses). The results were stratified into matches of the top diagnosis from the diagnostic agent and one of the top 3 diagnoses. These metrics were applied here to ChatGPT 3.5 and 4.0 and the WebMD SC.

Triage accuracy was calculated by comparing the triage level given by the diagnostic agent with the triage level of at least 2 of the 3 independent physicians. Triage was recorded as recommended levels of urgency of care: (1) home care, (2) routine primary care, and (3) urgent or emergency care. The independent physicians were asked for triage recommendations using a more detailed scale, which was then mapped to these 3 levels. ChatGPT has standard text for routine care: “It is recommended that the individual consults with a healthcare professional for a proper evaluation and diagnosis.” The response from WebMD varies by diagnosis. An example of routine care triage for the diagnosis of chronic kidney disease is: “See your doctor if you have any symptoms of chronic kidney disease such as fatigue, decreased urination, swelling of legs, nausea, headache, and weakness.” For urgent/emergency triage, ChatGPT outputs variations similar to the following text: “It is important for the patient to seek immediate medical attention,” while WebMD displays an alert at the top of the diagnosis page that reads: “This is an URGENT CONDITION. Please contact your doctor or call 911.”

This study design and the clinical data collected were used to evaluate the two versions of ChatGPT, versions 3.5 and 4.0. An RA (DC) entered the symptom data collected by the Ada SC for each case into ChatGPT and recorded the output. The RA was blinded to the actual diagnosis and triage results from the 2022 study [20]. For comparison, the Ada symptom data were also entered into the widely used SC from WebMD [25]. As with the previous study, diagnostic accuracy was evaluated by the number of cases where a ChatGPT or WebMD diagnosis matched at least one diagnosis from the ED physician who saw the patient. This was reported for matches for the top suggested diagnosis or any of the top 3 diagnoses. Matching was carried out by one author (HF) and then reviewed and verified by another author (RH), resulting in small changes to the overall scores.

The original study [20] used a version of Ada from 2018; however, the current version has undergone extensive updates, in part to qualify under EU regulations. As an approach to compare differences in the algorithms, we attempted to reenter data from the first 20 cases into the current version of Ada (released June/July 2023). The questions asked by Ada had been updated, resulting in modest differences in the questions regarding symptoms recorded as present, but large differences in questions regarding symptoms recorded as absent. It was therefore not possible to reliably compare the performance by this method; hence, only the original Ada performance is reported.

### Ethical Approval

The original study was approved by the Institutional Review Board, Research Data Protection Office, Lifespan Healthcare, Providence, Rhode Island (1439681-3). Only secondary deidentified data were used for the current study.

## Results

### Diagnostic Accuracy

Results for diagnostic accuracy are shown in Table 1. From the original study [20], Ada had a match rate between its top-1 diagnosis and the final ED diagnosis of 9 (30%). The top match rate for ChatGPT 3.5, ChatGPT 4.0, WebMD, and the physicians mean rate was 40% (n=12), 33% (n=10), 40% (n=12), and 47%, respectively. The top-3 diagnostic match rate for Ada, ChatGPT 3.5, ChatGPT 4.0, WebMD, and physicians mean rate was 63% (n=19), 63% (n=19), 50% (n=15), 57% (n=17), and 69%, respectively.

**Table 1:** Diagnosis results for Ada, ChatGPT, and WebMD (N=30).

Matches	Ada, n (%)	ChatGPT 3.5, n (%)	ChatGPT 4.0, n (%)	WebMD, n (%)
Top 1	9 (30)	12 (40)	10 (33)	12 (40)
Top 3	19 (63)	19 (63)	15 (50)	17 (57)

**Figure 2:**
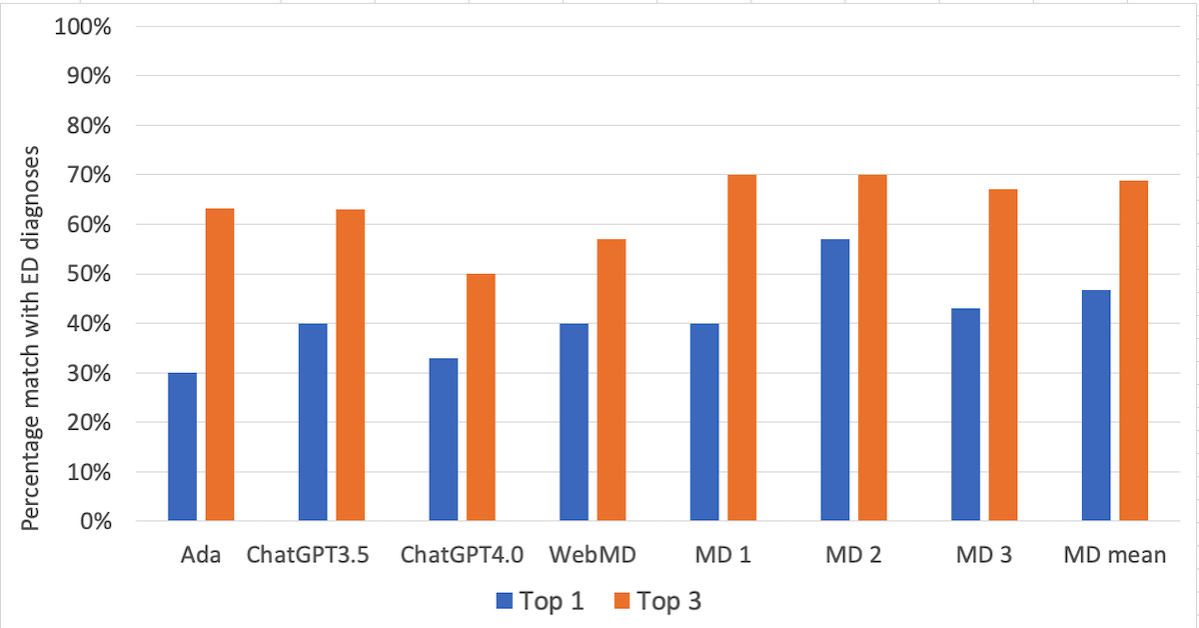
Diagnostic matches of Ada, ChatGPT 3.5 and 4.0, WebMD, and the independent emergency department (ED) physicians (MD 1, MD 2, MD 3). The gold standard is defined as diagnoses from ED discharge notes.

Figure 2 shows the comparison of the 4 diagnostic tools with the 3 independent ED physicians using the same symptom data. Because the ED physician actually saw the patient and carried out a physical examination and likely investigations, their diagnostic accuracy will likely be higher than can be achieved with the Ada symptom data alone.

ChatGPT 3.5 had the strongest combined diagnostic performance of the 4 systems, whereas ChatGPT 4.0 had the weakest combined performance. The differences between the results for top-1 and top-3 diagnosis matches between the different systems and the mean physician performance were not significant. We also examined any additional diagnosis matches at the rank of 4th or 5th in cases that did not already have a match. ChatGPT 3.5 had no additional matching diagnoses below the rank of 3, ChatGPT 4.0 had 3 additional matches, WebMD had 1, and Ada had 2. The physicians were only asked for 3 possible diagnoses. Most matches were clear, with less than one-quarter requiring any discussion, primarily to determine whether the level of specificity of the diagnoses matched. For example “Lower Back Herniated Disk” from ChatGPT 3.5 and “Lumbar (Low-Back) Herniated Disc” from WebMD were not considered a match with “acute bilateral back pain” from the ED physician, whereas “lumbar strain” from ChatGPT 4.0 was rated a match. Another issue was that ChatGPT 4.0 appeared to behave differently when prompted with “what is the diagnosis” than if the case was entered without the “what is the diagnosis” prompt. A broader differential was often generated without the prompt. This issue was not seen with ChatGPT 3.5.

### Triage Accuracy

As shown in Table 2, overall triage performance based on agreement between the systems and at least 2 of the 3 physicians was the highest for ChatGPT 4.0 (76%) and the lowest for ChatGPT 3.5 (59%). On the key statistic of unsafe triage, Ada performed the best with 14%, followed by WebMD with 19%, but ChatGPT 3.5 had much poorer performance at 41%. Comparing safe versus unsafe triage for Ada (5/37, 14%) and ChatGPT 3.5 (15/37, 41%), the difference was significant (*P*=.009; χ^2^ test). The difference between WebMD and ChatGPT 3.5 was not significant (*P*=.08) (Multimedia Appendix 1 shows the detailed triage results including 95% CIs). ChatGPT 3.5 had no examples of triage that were too cautious and had a high rate of missing serious conditions requiring urgent or emergency care. ChatGPT 4.0 showed different behavior to ChatGPT 3.5. Agreement with the physician reviewers was high at 76% (28/37), the rate of *unsafe* triage fell to 22% (8/37), and the rate of *too cautious* triage increased slightly to 3% (1/37). Between ChatGPT 3.5 and 4.0, one case changed from *agree* to *unsafe* and one changed from *agree* to *too cautious*.

**Table 2:** Triage accuracy of symptom checkers and ChatGPT compared to the assessment of independent emergency department physicians (N=37).

Triage assessment	Ada, n (%)	ChatGPT 3.5, n (%)	ChatGPT 4.0, n (%)	WebMD, n (%)
Agree	23 (62)	22 (59)	28 (76)	26 (70)
Unsafe	5 (14)	15 (41)	8 (22)	7 (19)
Too cautious	9 (24)	0 (0)	1 (3)	4 (11)

## Discussion

### Principal Findings

This study builds on a previously validated set of cases entered by actual patients presenting for emergency or urgent care. We believe these results provide a unique assessment of the performance of WebMD, ChatGPT 3.5, and ChatGPT 4.0 on real patient data, as opposed to vignettes created by physicians (as used in most other studies that have assessed the performance of ChatGPT). Differences between patient-reported data and physician-created vignettes may include patients’ medical knowledge or understanding of questions, the ability to distinguish symptoms not present versus unknown, and elicitation of unusual but potentially diagnostic symptoms. Additionally, the artificial limitation of most vignettes to point to a single diagnosis simplifies calculations of diagnostic accuracy but does not reflect most of the real clinical assessments seen in this study. A limitation here is that diagnostic accuracy is defined as sensitivity of the diagnostic agent’s output to at least one of the reviewing ED physicians’ diagnoses, rather than an assessment of the SC’s concordance with the full differential given by the ED physician. Comprehensiveness and relevance scores (which are similar to sensitivity and positive predictive value, respectively) can be used to account for the proportion of correct diagnoses matched by a diagnostic tool, which correlated with the diagnosis matching scores in the earlier Ada study [20].

Fully assessing diagnostic tools such as SCs requires that all the stages of use are evaluated: (1) patients’ ability to use the app, (2) ability of the app to collect a clinical history, (3) performance of the diagnostic and triage algorithms, and (4) patients’ ability to interpret and act on the results presented. The results presented here primarily address point (3), the performance of the diagnostic algorithm. Usability of the Ada app (1) was demonstrated in the earlier study [20]. Ada has also been shown to collect a more comprehensive history than most SC apps (2) [23]. In the similar study of Ada performed in a primary care setting, the independent physicians reviewing the symptom data of the 201 cases were asked to indicate any questions they considered were missing from the Ada-collected history. In 134 (67%) of the cases, 0 or 1 of the clinicians suggested additional questions (these results are in preparation for publication). WebMD might perform less well in direct use by patients as it typically collects less clinical data. The performance of ChatGPT as a history-taking tool in the hands of patients has yet to be determined. We are planning additional studies to address (4), the effect of the outputs of SCs and LLMs such as ChatGPT on patient decision-making in seeking care.

In the earlier study of Ada that generated the data used here [20], the handling of cases with symptoms recorded as the final ED diagnosis was reviewed by the research team. In total, 33 cases had full data to carry out diagnostic analysis, but in 3 cases, the ED diagnosis remained unclear despite a second chart review (2 cases had a chief complaint of abdominal pain and the other had a chief complaint of dizziness). These 3 cases were excluded from the diagnostic analysis but included in the triage analysis. In 6 cases, the patient had chest pain as a symptom and underwent a screen for acute myocardial infarction (AMI). In those cases, a diagnosis of AMI or unstable angina was considered correct even if the patient ultimately had a negative screen. Two cases had a “diagnosis” listed as back pain; they were included in the analysis due to the high frequency of patients with back pain seen in the ED without specific diagnoses. Previous studies have shown that “symptom-based discharge” is common in EDs [28]. The process of defining one or more “correct diagnosis” from the ED physician’s assessment and the EHR note they created was a significant challenge. It was previously noted that “In many cases, the ED physicians’ role was to exclude serious causes for the presenting symptoms, with the patient potentially seeking investigation through their primary care physician or specialist at a later date” [20].

The result of triage performance presented a mixed picture. Compared to Ada, WebMD had a higher *agree* level with the independent physicians (70%) but also had a higher *unsafe* rate of 19%. ChatGPT 3.5 had a slightly lower *agree* score than Ada and a much higher *unsafe* triage rate. For the urgent and serious cases seen in this study, such an unsafe triage rate could put patients at genuine risk, discouraging them from seeking needed care quickly. ChatGPT 4.0 had a significantly better triage performance with a higher rate of *agree* compared to the two SCs and to previous vignette studies [5,6]. The unsafe triage rate of ChatGPT 4.0 was still higher than that for Ada or WebMD but much lower than that for ChatGPT 3.5.

There are a range of systems and scoring systems available for triage (such as the Manchester Triage System used by Cotte et al [22] and the ESI [27] used at RIH), which makes comparison with other studies more difficult. The earlier study [20] tested the effect of adding vital sign data on physicians’ triage decisions, but the effect was small. Studies of how patients are currently using LLMs for medical advice would also strengthen the conclusions and recommendations obtained here for future development and use of these systems.

### Comparison With Other Studies

In 2015, Semigran et al [5] compared the performance of 23 available SCs with 45 case vignettes. The average SC sensitivity for the correct diagnosis for their top diagnosis was 35.5% and that for the top 3 diagnoses was 51%. Independent physicians achieved a sensitivity of 72% for their top diagnosis and 84.3% for the top 3 diagnoses [29]. The 3 best-performing apps had a top-1 sensitivity of 43%-50% and top-3 sensitivity of 67%-70%, which are slightly higher than those obtained in this study. A repeat of that study 5 years later [6] showed an improvement in average diagnosis performance (of apps tested in both studies) with top-1 sensitivity of 45.5%. The top-1 sensitivity for Ada in that study was higher at 53%. These vignette studies differ from the real cases seen here in having only one “correct” diagnosis.

These studies also assessed triage accuracy for the SC apps that provided the output. In the 2015 study of Semigran et al [5], the mean correct triage (match between app and vignette) rate was 59.1%. For the Schmieding et al [6] study performed 5 years later, the rate was slightly lower at 55.8%. However, the authors noted that the apps were less risk-adverse, resulting in undertriaging more than 40% of the vignettes rated as an emergency, which was a similar level to the result with ChatGPT shown here. Schmieding et al [6] used the 45 case vignettes (with simplified language) to evaluate the triage accuracy of lay users. They showed a correct triage rate of 60.9% for lay users compared to only 58% for SCs. They noted that “most lay participants outperformed all but 5 SCs.” Importantly, the SCs had higher scores for emergencies in this study but lower scores for low-risk cases. Three studies evaluated SC performance on actual emergency or urgent care patients. Cotte et al [22] (working for Ada) evaluated Ada’s triage accuracy on 378 “walk-in” patients in urgent care. They compared its triage accuracy with the result from the Manchester Triage System and showed an undertriage rate of 8.9% of cases and an overtriage rate of 57.1%. A study of 2 SCs based on chart review of 100 records in an ED in Hong Kong showed triage accuracies of 50% and 74%, but noted poorer performance on more urgent cases [30]. A study in Canada of a locally developed SC showed significantly better triage accuracy than patients (73% vs 58%; *P*<.01), with better performance on emergency cases [31].

In 2023, Levine et al [15] used the 48 case vignettes developed for a previous study of patients directly assessing diagnosis and triage [32]. They evaluated GPT-3, the underlying model of ChatGPT. The results showed that GPT-3 had the correct diagnosis from the vignette in its top 3 88% of the time compared to the correct rate of lay individuals of 54%, (*P*<.001). this figure was 75%, and for lay individuals 43%. For triage, GPT-3 had an *agree* rate of 70%, which was similar to that of lay individuals of 74%. They did not report unsafe triage rates. Unlike the present study, Levine et al [15] were able to evaluate the performance of GPT-3 on low-risk “self-care” cases and showed that triage accuracy was much lower at 50%. SC evaluation studies have also shown lower accuracy on self-care cases [6]. This is a particular concern regarding the ability of diagnostic tools to help reduce overload on urgent and emergency care systems. The authors also note that their vignettes are “simulated cases” and that the diagnostic agents “may perform differently when presented with real-world symptoms.” Another concern is that presenting data to ChatGPT in different ways may affect the output; therefore, if patients pose a question in a specific manner, they may receive less accurate results. More guidance is required regarding the standardization of medical queries and ideally specific entry modes for medical questions in LLMs.

A notable feature of the triage behavior of ChatGPT 3.5 shown here was that *in many cases it conflicted with the diagnoses it produced*. Cases rated as unsafe in this study included diagnoses that were correctly made for myocardial infarction, pyelonephritis, and head injury, yet urgent/emergency triage was not recommended. A simple triage rule that tied the minimum triage level to the most serious and urgent diagnoses listed would have the potential to correct these errors and reduce unsafe triage performance. Both Ada and WebMD appear to link diagnosis and triage levels in this way. Similar discordant results between diagnosis and triage have been seen with other SCs in a previous study [6].

The behavior of ChatGPT 4.0 was significantly different from that of version 3.5. There was a large improvement in triage performance, which at 76% was the highest agreement with the physicians’ triage seen in our study. This is also very high compared to the performances from previous vignette studies [5,6,33]. Unsafe triage also improved with the updated version of ChatGPT, but was still almost twice that of Ada. In traditional diagnostic algorithms (such as Bayesian networks used by Ada), the output threshold can be adjusted to favor higher or lower levels of urgency of care overall. ChatGPT 3.5 and 4.0 would likely benefit from such an adjustment, reducing *unsafe* triage and increasing the *too cautious* category. Given the generally high diagnostic accuracy seen with ChatGPT 3.5, safety and consistency of triage and confidence in the system would seem to require such “guard rails.” An important aspect of the performance of ChatGPT 4.0 was the substantial drop in diagnostic accuracy compared to that of version 3.5, which could make tying triage to diagnoses less effective. ChatGPT 4.0 also produced more examples of general diagnostic categories than found for version 3.5, such as “renal disease” rather than specific diseases that had strong matches to the ED diagnoses.

Chen et al [34] recently compared the performance of ChatGPT versions 3.5 and 4.0 on four standard problems both soon after the initial release of version 4.0 in March 2023 and again in June 2023. They reported large falls in performance on tasks such as math problems and programming with the newer version, but some improvements in the previous version. Both versions had more formatting mistakes in code generation in June. These differences were attributed to large changes in the LLM algorithms, which were made without notification to the users (who had paid a subscription for the version 4.0 service). We used ChatGPT 4.0 for the current ED study in June 2023 and may therefore have seen lower diagnostic and triage performance than studies carried out earlier. Gilbert et al [35] made an assessment of the potential regulation of LLMs as medical devices, particularly for tasks such as clinical decision support. For the categories of *Verification, Usability,* and *Surveillance*, they noted that the near-infinite range of possible inputs and outputs prevents standardized regulation of current approaches. For the categories of *Provenance* and *Changes,* they noted the lack of a stable controlled versioning (as also noted above) as a serious barrier. As there is no proven method currently available to prevent harmful outputs, they argue that current methods of *risk mitigation* are also ineffective. While LLMs used for decision support are required to be regulated as medical devices in the United States and European Union/United Kingdom [35], they do not seem to qualify in their current form. These concerns are all multiplied if the system is being used by patients without input or oversight from medical professionals.

### Limitations

This study did not include examples of patients that would likely not have required any clinical care (ie, could have managed their symptoms at home). Future studies need to evaluate the question of overtriage and the risk of increasing unnecessary visits to health facilities, an area our primary care study should help to address. Other limitations of this study include the relatively small number of cases available for measurement of diagnosis. This is being addressed in the ongoing 201-patient primary care study, along with newly funded studies being developed in the ED and primary care settings with a plan to recruit an additional 700 patients. Larger studies will also help address questions regarding the performance and usability of systems by different patient groups. The usability of the Ada app by patients assessed through the questionnaire in the ED and primary care studies did not detect evidence of differences by race, ethnicity, or gender. However, lower usability for patients aged over 60 years was seen.

Another possible limitation is that changing the wording of prompts to ChatGPT can make a significant difference to the system’s output. This has the potential to change measured diagnostic and triage performance. To address this, we intentionally used a simple approach to entering the data and simply prompted with “What is the diagnosis” to try and capture the likely behavior of patients. This prompt is similar to that used in the previous study of patient diagnosis and health information–seeking behavior by Levine and colleagues [32]. Further study is required to evaluate the effects of different prompts; as noted in the Results, behavior was different with ChatGPT 3.5 and 4.0. It is our view that the developers of ChatGPT (Open AI Inc and Microsoft Inc) and other LLMs have a responsibility to provide clear instructions, accept a range of prompts, and query the user if their intention is not clear, rather than place the responsibility and risk on the patient.

### Conclusions

LLMs such as ChatGPT are a new technology with surprising performance in the analysis of medical data, including the provision of medical diagnosis and triage. The ease of use of these tools and the high quality and appealing textual output they produce are likely to make them attractive to people seeking answers in many areas, including medicine and health. As has been seen with the use of search engines such as Google [36] and existing SCs [7,20], a substantial proportion of the population are likely to use such tools. Now that ChatGPT is built into the search engine Bing (Microsoft Inc), it is likely to see much greater use. The results of this study show both the important potential of LLMs like ChatGPT and a number of serious concerns about their current performance and safety. In future studies, we plan to include other LLMs such as Bard and the medically focused example Med-PaLM, both from Google Inc. Rigorous clinical evaluation of this sort with a wide range of real patients and different types of potential illnesses is essential to prevent these exciting and attractive tools from achieving widespread and unregulated use without a clear understanding of the risks. Otherwise, it is likely they will provide unsafe advice to a subset of patients with potentially life-threatening conditions and increase unnecessary visits to urgent or emergency care for less serious conditions.

## Appendix

Multimedia Appendix 1 Detailed results of the triage matches shown in Table 2, including 95% CIs.

## Abbreviations

AMI: acute myocardial infarction
ED: emergency department
EHR: electronic health record
ESI: Emergency Severity Index
LLM: large language model
RA: research assistant
RIH: Rhode Island Hospital
RLHF: reinforcement learning with human feedback
SC: symptom checker

## References

[1] Hamilton W (2012). Diagnosis: Cancer diagnosis in UK primary care. Nat Rev Clin Oncol.

[2] Vardell E, Moore M (2011). Isabel, a clinical decision support system. Med Ref Serv Q.

[3] Barnett GO, Cimino JJ, Hupp JA, Hoffer EP (1987). DXplain. An evolving diagnostic decision-support system. JAMA.

[4] Shortliffe EN, Shah NH, Cohen TA, Patel VL, Shortliffe EH (2022). AI in medicine: some pertinent history. Intelligent systems in medicine and health. The role of AI.

[5] Semigran H, Linder JA, Gidengil C, Mehrotra A (2015). Evaluation of symptom checkers for self diagnosis and triage: audit study. BMJ.

[6] Schmieding ML, Kopka M, Schmidt K, Schulz-Niethammer S, Balzer F, Feufel MA (2022). Triage accuracy of symptom checker apps: 5-year follow-up evaluation. J Med Internet Res.

[7] Gilbert S, Mehl A, Baluch A, Cawley C, Challiner J, Fraser H, Millen E, Montazeri M, Multmeier J, Pick F, Richter C, Türk E, Upadhyay S, Virani V, Vona N, Wicks P, Novorol C (2020). How accurate are digital symptom assessment apps for suggesting conditions and urgency advice? A clinical vignettes comparison to GPs. BMJ Open.

[8] Hardesty L (2017). Explained: neural networks. MIT News.

[9] (2023). What is ChatGPT? Commonly asked questions about ChatGPT. OpenAI.

[10] Akyurek E (2023). What learning algorithm is in-context learning? Investigations with linear models. arXiv.

[11] Zewe A (2023). Solving a machine-learning mystery. MIT News.

[12] Harskamp RE, De Clercq L (2023). Performance of ChatGPT as an AI-assisted decision support tool in medicine: a proof-of-concept study for interpreting symptoms and management of common cardiac conditions (AMSTELHEART-2). medRxiv.

[13] Thirunavukarasu AJ, Hassan R, Mahmood S, Sanghera R, Barzangi K, El Mukashfi M, Shah S (2023). Trialling a large language model (ChatGPT) in general practice with the applied knowledge test: observational study demonstrating opportunities and limitations in primary care. JMIR Med Educ.

[14] Gilson A, Safranek CW, Huang T, Socrates V, Chi L, Taylor RA, Chartash D (2023). How does ChatGPT perform on the United States medical licensing examination? The implications of large language models for medical education and knowledge assessment. JMIR Med Educ.

[15] Levine D, Tuwani R, Kompa B, Varma A, Finlayson SG, Mehrotra A, Beam A (2023). The diagnostic and triage accuracy of the GPT-3 artificial intelligence model. medRxiv.

[16] Cascella M, Montomoli J, Bellini V, Bignami E (2023). Evaluating the feasibility of ChatGPT in healthcare: an analysis of multiple clinical and research scenarios. J Med Syst.

[17] Benoit JR (2023). ChatGPT for clinical vignette generation, revision, and evaluation. medRxiv.

[18] Alkaissi HS, McFarlane SI (2023). Artificial hallucinations in ChatGPT: implications in scientific writing. Cureus.

[19] Fraser H, Coiera E, Wong D (2018). Safety of patient-facing digital symptom checkers. Lancet.

[20] Fraser HSF, Cohan G, Koehler C, Anderson J, Lawrence A, Pateña J, Bacher I, Ranney ML (2022). Evaluation of diagnostic and triage accuracy and usability of a symptom checker in an emergency department: observational study. JMIR Mhealth Uhealth.

[21] (2022). Ada Health.

[22] Cotte F, Mueller T, Gilbert S, Blümke B, Multmeier J, Hirsch MC, Wicks P, Wolanski J, Tutschkow D, Schade Brittinger C, Timmermann L, Jerrentrup A (2022). Safety of triage self-assessment using a symptom assessment App for Walk-in Patients in the emergency care setting: observational prospective cross-sectional study. JMIR Mhealth Uhealth.

[23] Ceney A, Tolond S, Glowinski A, Marks B, Swift S, Palser T (2021). Accuracy of online symptom checkers and the potential impact on service utilisation. PLoS One.

[24] Montazeri M, Multmeier J, Novorol C, Upadhyay S, Wicks P, Gilbert S (2021). Optimization of patient flow in urgent care centers using a digital tool for recording patient symptoms and history: simulation study. JMIR Form Res.

[25] WebMD symptom checker.

[26] Harris PA, Taylor R, Thielke R, Payne J, Gonzalez N, Conde JG (2009). Research electronic data capture (REDCap)--a metadata-driven methodology and workflow process for providing translational research informatics support. J Biomed Inform.

[27] Emergency Severity Index (ESI): a triage tool for emergency departments. Agency for Healthcare Research and Quality.

[28] Slovis B, McCarthy D, Nord G, Doty A, Piserchia K, Rising K (2019). Identifying Emergency Department Symptom-Based Diagnoses with the Unified Medical Language System. West J Emerg Med.

[29] Semigran HL, Levine DM, Nundy S, Mehrotra A (2016). Comparison of physician and computer diagnostic accuracy. JAMA Intern Med.

[30] Yu SWY, Ma A, Tsang VHM, Chung LSW, Leung S, Leung L (2019). Triage accuracy of online symptom checkers for accident and emergency department patients. Hong Kong J Emerg Med.

[31] Chan F, Lai S, Pieterman M, Richardson L, Singh A, Peters J, Toy A, Piccininni C, Rouault T, Wong K, Quong JK, Wakabayashi AT, Pawelec-Brzychczy A (2021). Performance of a new symptom checker in patient triage: Canadian cohort study. PLoS One.

[32] Levine DM, Mehrotra A (2021). Assessment of diagnosis and triage in validated case vignettes among nonphysicians before and after internet search. JAMA Netw Open.

[33] Hill MG, Sim M, Mills B (2020). The quality of diagnosis and triage advice provided by free online symptom checkers and apps in Australia. Med J Aust.

[34] Chen L, Zharia M, Zou J (2023). How is ChatGPT's behavior changing over time?. arXiv.

[35] Gilbert S, Harvey H, Melvin T, Vollebregt E, Wicks P (2023). Large language model AI chatbots require approval as medical devices. Nat Med.

[36] Millenson ML, Baldwin JL, Zipperer L, Singh H (2018). Beyond Dr. Google: the evidence on consumer-facing digital tools for diagnosis. Diagnosis.

