# Supplementary material for: Comparison of Diagnostic and Triage Accuracy of Ada Health and WebMD Symptom Checkers, ChatGPT, and Physicians for Patients in an Emergency Department: Clinical Data Analysis Study
Source: JMIR Mhealth Uhealth. 2023 Oct 3;11:e49995. doi: 10.2196/49995 (PMC10582809; doi:10.2196/49995)
Supplement: Multimedia Appendix 1 [file mhealth_v11i1e49995_app1.pdf]

Appendix 1
Triage matches from Table 2 including 95% Confidence Intervals

Calculated using the proportions method. N=37 cases.

Triage Level	System	Cases	Match (95% CI)
Unsafe	Ada	5	13.5% (2.5% - 24.5%)
	ChatGPT v3.5	15	40.5% (24.7% - 56.4%
	ChatGPT v4.0	8	21.6% (8.4% - 34.9%)
	WebMD	7	18.9% (6.3% - 31.5%)
Agree	Ada	23	62.2% (46.5% - 77.8%)
	ChatGPT v3.5	22	59.5% (43.6% - 75.3%)
	ChatGPT v4.0	28	75.7% (61.9% - 89.5%)
	WebMD	26	70.3% (55.5% - 85.0%)
Too Cautious	Ada	9	24.3% (10.5% - 38.1%)
	ChatGPT v3.5	0	0% (0% - 0%)
	ChatGPT v4.0	1	2.7% (0% - 7.9%)
	WebMD	4	10.8% (0.8% - 20.8%)

Comparing safe versus unsafe triage for Ada (5/37, 14%) and ChatGPT 3.5 (15/37, 47%), the difference was significant (P=.009; $\chi 2$ test).

Comparing safe versus unsafe triage for WebMD and ChatGPT 3.5 (15/37, 47%), (19/37) was not significant (P=.08; $\chi 2$ test).
